# Chest X-rays findings in COVID 19 patients at a University Teaching Hospital - A descriptive study

**DOI:** 10.12669/pjms.36.COVID19-S4.2778

**Published:** 2020-05

**Authors:** Misbah Durrani, Inam ul Haq, Ume Kalsoom, Anum Yousaf

**Affiliations:** 1 Dr. Misbah Durrani, MCPS. FCPS. Department of Diagnostic Radiology. Benazir Bhutto Hospital, Rawalpindi, Pakistan; 2 Dr. Inam ul Haq, FCPS. MHPE. Al-Shifa Trust Eye Hospital, Rawalpindi, Pakistan; 3 Dr. Ume Kalsoom FCPS. Department of Diagnostic Radiology. Benazir Bhutto Hospital, Rawalpindi, Pakistan; 4 Dr. Anum Yousaf, Resident. Department of Diagnostic Radiology. Benazir Bhutto Hospital, Rawalpindi, Pakistan

**Keywords:** COVID-19 patients, Chest X-rays (CXR), Corona virus, British Society of thoracic Imaging classification (BSTI)

## Abstract

**Objective:**

To analyze Chest X-ray findings in COVID 19 positive patients, presented at corona filtration center, Benazir Bhutto Hospital Rawalpindi, based on CXR classification of British Society of Thoracic Imaging (BSTI).

**Methods:**

In this study, all RT-PCR COVID-19 positive patients screened at corona filtration center, Benazir Bhutto hospital Rawalpindi from 20^th^ March 2020 to 10^th^ April 2020 were included. Mean age of the cohort with age range was calculated. Presenting complaints & Co-morbid were analyzed and tabulated in frequencies and percentages. Portable CXR findings were classified according to BSTI classification and documented in frequencies and percentages.

**Results:**

Mean age of the patients was 44 years. Presenting complaints were cough 20 (67%), fever 18 (60%), shortness of breath 11 (37%), sore throat six (20%), loss of sense of taste and smell four(13%). Main co-morbid was hypertension six (20%). Two (7%) patients had normal and seven (23%) had classical COVID CXRs. 21 (70%) patients were in indeterminate group with only one (3%) having unilateral lung disease. Three (10%) patients had diffuse lung involvement and 18(60%) had peripheral lung involvement. Majority of patients 19 (63%), had bilateral middle and lower zonal involvement.

**Conclusions:**

In this study, COVID-19 CXRs generally manifested a spectrum of pure ground glass, mixed ground glass opacities to consolidation in bilateral peripheral middle and lower lung zones. BSTI CXR reporting classification of COVID-19 is valid in our patients with addition of middle zonal involvement in classical COVID-19 criteria as opposed to just lower zone involvement.

## INTRODUCTION

Devastations of COVID 19 are revealing day by day. This pandemic has spread all across the globe. This disease is still a mystery. We are not able to comprehend; why this disease has different out comes in different individuals irrespective of age, race and regional constraints. Spectrum of COVID 19 positive patients varies from asymptomatic to patient on ventilators and ultimately death.

Radiological evaluation is one way of looking in the body.^1^ CXRs are the most commonly performed investigation in COVID 19 suspected cases.^2^ British Society of Thoracic Imaging (BSTI) has classified CXRs findings, based on European patients.^3^ Our local population is different both in habitat and disease patterns, it was therefore thought to see pattern of CXR findings in our COVID 19 positive patients. CT scan is a preferred investigation in terms of diagnosis and disease follow up, but it is not feasible to use it as a screening tool in terms of its availability and rigorous time consuming decontamination measures.

The purpose of this study was to analyze chest X-rays findings in our patients based on British Society of Thoracic Imaging classification and to evaluate disease pattern in terms of any deviation or similarity. Another rationale is that X-ray facilities are available in basic health units and this study will enhance our clinicians understanding of CXR findings in suspected COVID 19 patients.

## METHODS

This is a retrospective descriptive study conducted at Isolation ward BBH hospital Rawalpindi. All RT-PCR COVID 19 positive patients presented in corona filtration center Benazir Bhutto hospital from 20 March 2020 to 10 April 2020 were included. Benazir Bhutto hospital Rawalpindi is a teaching hospital affiliated with Rawalpindi Medical University”. It has a designated COVID-19 filter clinic where suspected patients are screened initially with portable chest x- rays and RT-PCR throat swab. Chest X-rays are stored in central computer server of Benazir Bhutto hospital. Chest radiographs of all COVID-19 confirmed patients from 20th March 2020 to 10th April 2020 were included in this study, irrespective of age or gender and were classified according to BSTI classification.

### Data Analysis and results

Quantitative variables like age is presented as mean along with age range. Qualitative variables like gender and co morbid were presented as frequency and percentages. Outcome variable, portable CXR findings were presented as frequency and percentages.

## RESULTS

Thirty COVID 19 positive cases had reported during the specified time, all were included in the study. Mean age of the patients was 44 with age range 7- 81 years. Two patients were of seven years of age. There were 24 (80%) males and six (20%) females. Male predilection of this disease is noted down in this study. 10 (33%) patients had history of travel. History of contact was positive in only five (17%) patients. Cough was the predominant presenting complaint in 20 (67% patients, followed by fever in 18 (60%), Shortness of breath 11 (37%), sore throat six (20%), loss of sense of smell and taste four (13%) and GIT complaints in three (10%) patients. In eight (27%) patients there were no co morbid. Six (20%) patients had ischemic heart disease and hypertension, three (10%) patients had diabetes, two (7%) patients were smoker, and six (18%) patients had other diseases tabulated in Table-I.

**Table-I:** Patient’s profile.

S. No			Findings
1	Age	Mean	44 years
		Range	7-81 years
2	Gender	Male	*n* (%)
			24(80%)
		Female	6(20%)
3	H/O Travel		10(33%)
4	H/O Contact		5 (17%)
5	Symptoms	Cough	20(67%)
		Fever	18(60%)
		Shortness of breath	11(37%)
		Sore throat	6(20%)
		Loss of sense of taste and smell	4(13%)
		GIT symptoms	3(10%)
6	Co-morbid	No co-morbid	8(27%)
		IHD & Hypertension	6(20%)
		Diabetes Mellitus	3(10%)
		Smoker	2(7%)
		Renal complaints	1(3%)
		stroke	1(3%)
		Tuberculosis	1(3%)
		Asthma	1(3%)
		Arthritis	1(3%)
		Malignancies	1(3%)

(Figures are presented as whole numbers with percentages in brackets).

Chest X-rays of all thirty patients were classified as normal, classical and indeterminate according to BSTI COVID-19 CXR classification. Two patients had normal chest X-rays (7%) and seven patients (23%) had classical picture of bilateral peripheral, basal ground glass haze/consolidation. Rests of twenty-one patients (70%) were falling in indeterminate group with one (3%) having unilateral lung disease and 20 (67%) patients had bilateral lung disease. Diffuse lung involvement was seen in three (10%) and peripheral lung involvement in 18 (60%) of patients. Majority of indeterminate patients, 19 (63%) had bilateral middle and lower zonal involvement and only two (7%) patients had middle zone involvement. Associated features in indeterminate group were pleural effusion four (13%), old healed calcific granulomas one (3%), and bilateral hilar lymphadenopathy one (3%). There were no cavitating lesions or pneumothorax. Table-II.

**Table-II:** Radiographic findings using BSTI COVID-19 CXR report proforma in patients.

Findings	No of patients
1.NORMAL correlated with RT-PCR	2 (7%)
2.CLASSIC /PROBABLE COVID -19 Consolidation /ground glass haze	
• Bilateral, peripheral, basal	7 (23%)
Indeterminate for COVID -19 • Consolidation/ground glass haze	
i). Location	
Unilateral	1 (3%)
Bilateral	20 (67%)
ii). Distribution	
Diffuse lung involvement	3 (10%)
Peripheral lung involvement	18 (60%)
iii). Zonal predominance	
Middle and lower zones involvement	19 (63%)
Only Middle zones involvement	2 (7%)
• Associated Features	
Pleural Effusion	4 (13%)
Old healed calcific granulomas	1 (3 %)
Cavitating lesions/pneumothorax	0 (0 %)
Bilateral hilar lymphadenopathy	1 (3 %)

(Figures are presented as whole numbers with percentages in brackets).

## DISCUSSION

Viruses belonging to the family of *coronavirdae* had already resulted in acute respiratory distress syndrome (SARS) in 2003 and Middle East respiratory syndrome^4^,^5^ (MERS) in 2012. COVID 19 virus has recently erupted and is still a mystery. Lot of research is going on all across the world and knowledge is being shared. Portable chest X-ray is the most commonly performed radiological investigation in terms of feasibility and cost effectiveness even in developed countries. In a dedicated corona filtration centre like Benazir Bhutto hospital Rawalpindi where on an average more than hundred suspected patients are being screened for COVID 19, portable chest X-ray is the optimal radiological screening tool. Strict decontamination measures could be ensured which are not possible in busy general OPD X-ray rooms. Due to limited RT-PCR kits and delayed results up to 48 hours, cases of high clinical suspicion with positive CXR findings are kept in isolation wards. CXR has a low sensitivity^6^ and it is difficult to distinguish between COVID 19 and other viral pneumonias purely on CXR findings. CT scan is the preferred imaging modality regarding early detection of disease as well as of its complications but it has infection control challenges including strict decontamination measures, ventilation and airflow.^7^ In HY Yoon et al study,^8^ 33% patients had abnormal initial radiographic findings in contrast to 93% abnormal chest findings in our study. In SARS^9^ these initial abnormal chest findings were in 78.3-82.4% and in MERS^10^ 83.6%. In Wong HYF et al.^11^ study consolidation was found in 47% of cases and this finding is consistent with other studies.^12^

There is no published study in literature as yet regarding COVID -19 CXR findings in Pakistan, however there are lots of studies on Chinese, Korean, American and European population. British society of thoracic imaging^13^ has classified COVID -19 chest X-rays as normal co related with RT-PCR,^14^ classical, having multiple bilateral, peripheral basal opacities more bilateral than unilateral, indeterminate that does not fit into classical or non COVID descriptors and Non COVID-19 X-rays having pneumothorax, pleural effusion and pulmonary edema. UCLA CXR COVID reporting classification is based upon recommendation from Radiological society of North America^15^ as typical having multifocal peripheral opacities with differential diagnosis of drug toxicity, influenza pneumonia and organizational pneumonia, indeterminate as non peripheral consolidation with differential of lots of infectious processes, atypical with uncommon imaging features and negative (does not exclude COVID-19).

**Fig.1:**
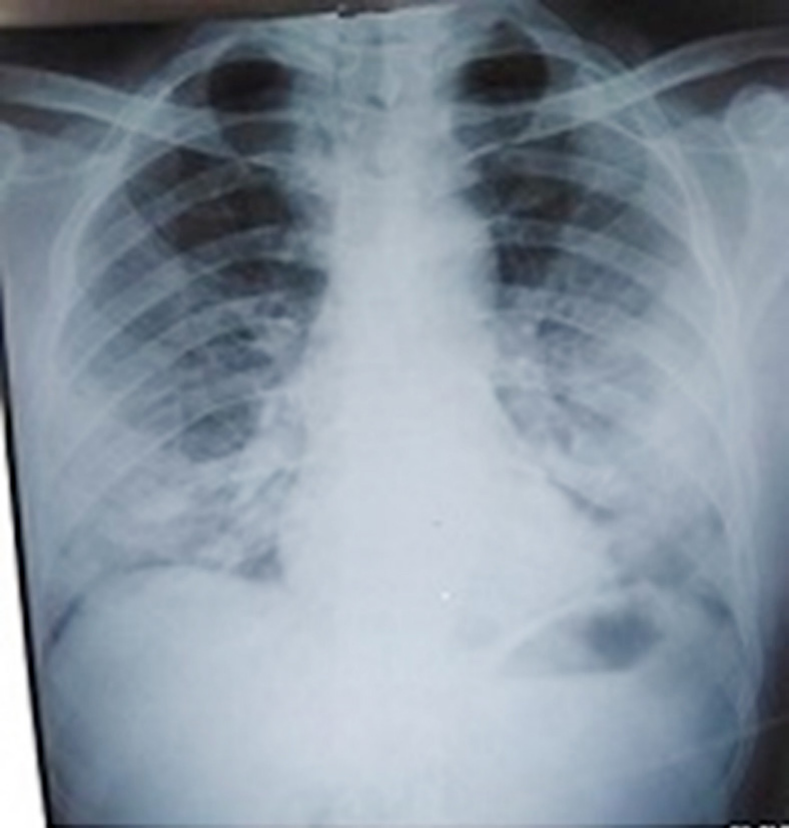
Chest X-ray of a seven years old female child showing Extensive Bilateral peripheral consolidation with air bronchograms predominantly right side, partially obscured right diaphragmatic silhouette and obscured ipsilateral CP angle. Radiographic findings are indeterminate for COVID-19. She had strong contact history, presented in ER with acute complains of SOB, cough, fever and flu.

Radiological findings were described according to Fleischner Society glossary of terms for Thoracic imaging.^16^ Ground glass opacities were defined as increased opacification of lung parenchyma not obscuring blood vessels and bronchi. Consolidation was described as homogenous opacification of lung parenchyma obscuring blood vessels and bronchi. We classified all CXRs on BSTI classification and found that majority of patients had bilateral, peripheral ground glass opacities and consolidation as documented in international studies. There may be diffuse lung involvement with perihilar infiltrates as well, marking severity of disease process. Fig.1.

**Fig.2:**
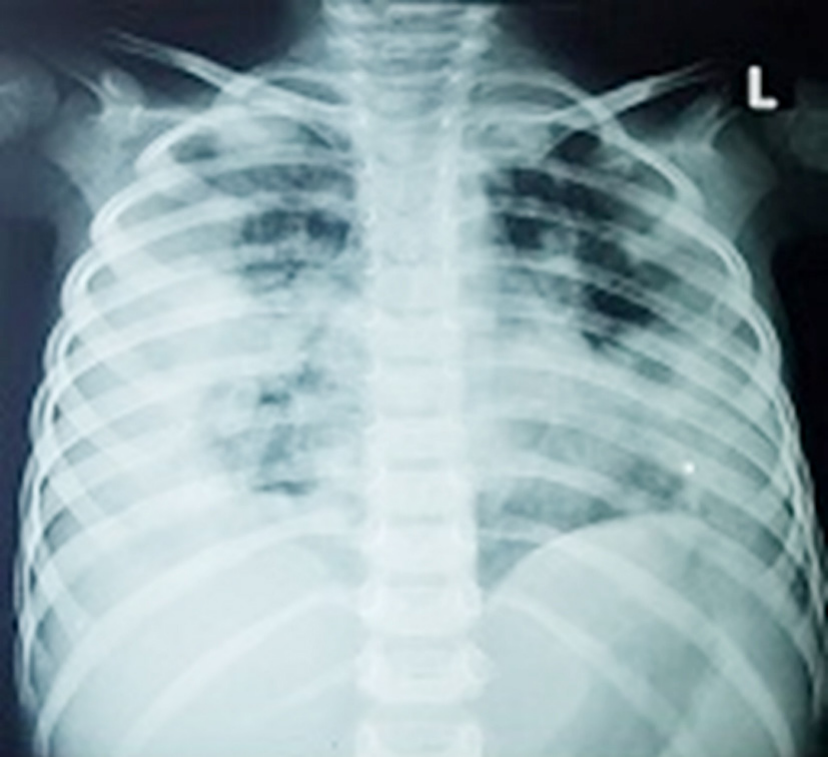
Chest X-ray image of a COVID -19 positive 56 years old male with no travel/contact history, presented with high grade fever, sore throat and cough for two days, showing bilateral mid and lower zones homogenous consolidation in peripheral distribution (L>R) along with Obscuration of both CP angles- Findings fall in the category of indeterminate for COVID-19.

Our study also shows that only seven (23%) of patients had BSTI classical picture of COVID -19 pneumonia of bilateral peripheral basal consolidation /ground glass haze. Majority of patients were of indeterminate group because of bilateral peripheral, multifocal middle and lower zonal lung involvement. Fig.2. This can imply that radiographic presentation of our patients was more severe in intensity.^17^

This pattern of consolidation had variable presentation in terms of shape and density. Some patients had smooth homogenous consolidation, while majority had inhomogenous, confluencing or patchy nodular opacities in peripheral distribution. Three patients with diffuse lung involvement had severe disease with no cephalocaudal or peripheral versus central discernment. Inclusion of middle lobe involvement in classical CXR definition of COVID -19 should be considered for Pakistani population. This could be a roadmap for future studies on a larger scale. There was no literature available from our neighboring countries to see if there was any similarity in classical CXR presentation of COVID -19 in South Asian population.

Indeterminate group included radiological characterization of COVID-19 keeping in view, peculiar presence of tuberculosis, seasonal emergence of allergic chest diseases and hypersensitivity pneumonitis in our local population. Pulmonary edema, interstitial pneumonitis and drug induced pneumonitis in immune-compromised patients can also mimic COVID-19 pneumonia. Uncommon imaging features such as lymph adenopathy and pleural effusion as mentioned in international studies were also uncommon in our local population.

### Limitations of the study

It included small sample size of just thirty patients as they were collected in initial twenty days of pandemic in our country. Now with increasing number of COVID positive patients all over Pakistan, a more comprehensive study should follow. Novelty of this early study of CXR finding of COVID-19 in Pakistan outweighed this limitation. Another limitation was absence of serial CXRs to see progression of disease, variable presentations and long term outcomes in our population. Clinical significance of this study is to provide an early insight into CXR finding of our patients. Portable chest X-rays^18^ are most readily available and feasible investigation in our urban and rural set ups. Its additional advantage is that there are minimal chances of cross contamination. Therefore, our general practitioners, clinicians, radiologists and paramedical health care workers can all benefit from findings of this study.

## CONCLUSION

COVID-19 pneumonia generally manifested a spectrum of pure ground glass, mixed ground glass opacities to consolidation in bilateral peripheral middle and lower lung zones in our local population. BSTI chest reporting classification COVID-19 is valid in our patients with addition of middle zonal involvement in classical COVID-19 criteria as opposed to just lower zone involvement.

### Authors’ Contribution

**MD:** Conceived, designed, manuscript writing and final review.

**UK & AY:** Did data collection and manuscript writing.

**IUH:** Did statistical analysis and editing of manuscript.
